# Data for analyzing drilling fluid ability to effectively achieve hole cleaning for high shear and low shear rates

**DOI:** 10.1016/j.dib.2018.06.007

**Published:** 2018-06-13

**Authors:** Adetola Solomon Adenubi, Kevin Chinwuba Igwilo, Emeka Emmanuel Okoro, Angela Onose Mamudu

**Affiliations:** a Petroleum Engineering Department, Covenant University Ota, Nigeria; b Chemical Engineering Department, Covenant University Ota, Nigeria

## Abstract

Rheological models such as Bingham Plastic or Power law models depict fluid behavior with points of the rheological relation which correspond to higher shear rates, but these models are fairly easy to solve for their specific descriptive parameters. Lower rpm (and hence shear rate), could be used to improve the performance and understanding of drilling mud at the lower shear rates prevailing in the wellbore. These data can be utilized in validating these rheological models and the essence of Equivalent Circulating Density (ECD) calculation in analyzing pressure drop in annular hole cleaning.

**Specifications Table**

**Table:** 

Subject area	*Petroleum Engineering*
More specific subject area	*Drilling and Well Engineering*
Type of data	*Table, graph, figure*
How data was acquired	*Experimental, Field data*
Data format	*Raw, analyzed*
Experimental factors	*The calculation of Equivalent Circulation Density (ECD) is dependent on accurate estimation of the annular pressure losses from the pressure gradient derived using these rheological models.*
Experimental features	*To analyze the various rheological models and attempt to identify an acceptable model for better appreciation of the annular pressure loss. Thus, derive the calculation of ECD while optimizing annular hole cleaning.*
Data source location	*Ota, Ogun State, Nigeria*
Data accessibility	*Data are available within this article*
Related research article	*None*

**Value of the data**

• The data can be applied in developing and validating a formula that will optimize hole cleaning during drilling operations.
• The data can be used to develop annular pressure loss model.
• The data can be utilized in obtaining direct model for the calculation of Equivalent Circulation Density.
• The data can be used to compare and justify the advantages and disadvantages of existing rheological models.

## 1 Data

Good hole cleaning practically depends on the type of weighting material and the model applied during drilling operation, [1], [2]. The laboratory experiments data for the drilling fluid at different weighting agent concentration and field data are tabulated in Table 1.

**Table 1:** Mud properties using calcium carbonate as the weighting material and field data.

**Activity**	**Standard**	**30 g Carbonate**	**60 g Carbonate**	**90 g Carbonate**	**120 g Carbonate**
***Drilling Mud Experimental Data***					
600 rpm	56	62	74	86	106
300 rpm	45	47	53	64	76
200 rpm	39	42	47	51	65
100 rpm	28	30	34	39	43
6 rpm	19	20	26	29	38
3 rpm	15	22	24	32	15
Flow Rate, Q gpm	854	854	854	854	854
Mud Weight, ppg	8.7	9.1	9.4	10.2	10.7
Plastic Viscosity (PV), cP	11	15	21	22	30
Yield Point (YP), lb/100 ft^2^	34	32	32	42	46
10 s Gel, lb/100 ft^2^	25	21	28	31	43
10 min Gel, lb/100 ft^2^	49	43	49	62	78
***Well Field Data***					
TVD, ft	2056	2056	2056	2056	2056
Length of Drill Collar (L_DC_), ft	150	150	150	150	150
Length of Drill Pipe (L_DP_), ft	2074	2074	2074	2074	2074
Length of Casing (L_Csg_), ft	2156	2156	2156	2156	2156
Hole Internal Diameter, ft	16	16	16	16	16
Drill-pipe Outer Diameter, ft	5.5	5.5	5.5	5.5	5.5
Drill-collar Outer Diameter, ft	7.785	7.785	7.785	7.785	7.785
Casing Internal Diameter, ft	17.570	17.570	17.570	17.570	17.570

**Fig. 1:**
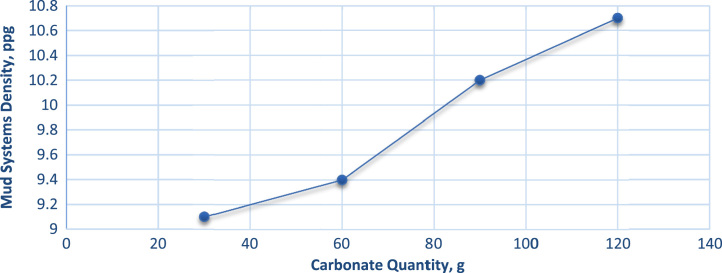
Effect of carbonate weighting agent concentration on mud system density.

**Fig. 2:**
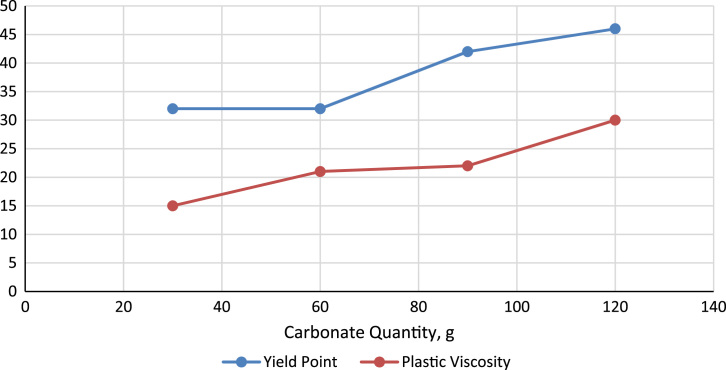
Effect of carbonate weighting agent concentration on Mud System Yield Point and *Plastic Viscosity*.

Given the data in Table 1, we calculated the pressure gradient, pressure loss and ECD using some rheological models for each of the weighting materials (Table 2, Table 3). Fig. 1 illustrates the effect of Carbonate concentration on mud density while Fig. 2. Illustrate its effect on yield point and plastic viscosity [3], [4].

**Table 2:** Calculated data from experimental and field data using Bingham Plastic Model.

Activity	**30 g Carbonate**	**60 g Carbonate**	**90 g Carbonate**	**120 g Carbonate**	**Standard**
***Pressure Loss along Drill Pipe in Casing***					
Drill-pipe Outer Diameter, ft	5.5	5.5	5.5	5.5	5.5
Casing Internal Diameter, ft	17.570	17.570	17.570	17.570	17.570
Drill-pipe Length, ft	2074	2074	2074	2074	2074
Total Pressure Loss, ΔP, psi	27.760	27.868	36.477	40.056	29.407
***Pressure Loss along Drill Pipe in Open Hole***					
Drill-pipe Outer Diameter, ft	5.5	5.5	5.5	5.5	5.5
Hole Internal Diameter, ft	16.0	16.0	16.0	16.0	16.0
Length of Drill-pipe, ft	82	82	82	82	82
Total Pressure Loss, ΔP, psi	1.267	1.274	1.665	1.831	1.340
***Pressure Loss along Drill Collars***					
Drill-collar Outer Diameter, ft	7.785	7.785	7.785	7.785	7.785
Hole Outer Diameter, ft	16.0	16.0	16.0	16.0	16.0
Drill-collar Length, ft	150	150	150	150	150
Total Pressure Loss, ΔP, psi	2.981	3.005	3.922	4.319	3.148
Equivalent Circulating Density	9.40	9.70	10.59	11.13	9.02

**Table 3:** Calculated data from experimental and field data using Power Law Model.

Activity	**30 g Carbonate**	**60 g Carbonate**	**90 g Carbonate**	**120 g Carbonate**	**Standard**
***Pressure Loss along Drill Pipe in Casing***					
Drill-pipe Outer Diameter, ft	5.5	5.5	5.5	5.5	5.5
Casing Internal Diameter, ft	17.570	17.570	17.570	17.570	17.570
Drill-pipe Length, ft	2074	2074	2074	2074	2074
Total Pressure Loss, ΔP, psi	0.134	0.178	0.198	0.243	0.131
***Pressure Loss along Drill Pipe in Open Hole***					
Drill-pipe Outer Diameter, ft	5.5	5.5	5.5	5.5	5.5
Hole Internal Diameter, ft	16.0	16.0	16.0	16.0	16.0
Length of Drill-pipe, ft	82	82	82	82	82
Total Pressure Loss, ΔP, psi	0.007	0.008	0.009	0.011	0.006
***Pressure Loss along Drill Collars***					
Drill-collar Outer Diameter, ft	7.785	7.785	7.785	7.785	7.785
Hole Outer Diameter, ft	16.0	16.0	16.0	16.0	16.0
Drill-collar Length, ft	150	150	150	150	150
Total Pressure Loss, ΔP, psi	0.016	0.021	0.023	0.027	0.016
Equivalent Circulating Density	9.10	9.40	10.20	10.70	8.70

## 2 Experimental design, materials, and methods

The laboratory work was carried out following the API standard and the materials used are tabulated in Table 4. The rheological tests which is the function of the hole cleaning and their weight properties were carried out using V-G meter and the mud balance as the measuring equipment [6], [8].

**Table 4:** Materials and additives used in formulating the water based muds (WBM).

**Product name**	**Mixing order & time**	**Brand name**	**Product specific gravity**	**Product concentration field barrel**		**Product concentration lab barrel**	
	**Min**			**Lbs/Bbl**	**Gals/Bbl**	**Grams**	**Mils**
WATER	0	Water	1	251.00		251.00	342.94
Viscosifier 2	2		1.5	1.50		1.50	1.00
Fluid loss Additive 1	1	LV	2	0.15		1.25	1.25
Alkalinity		Soda Ash	2.5	0.25		0.25	0.10
NACL	2	NACL	3.31	14.54		14.54	4.37
Other	1	Caustic Soda	2.13	0.25		0.25	0.12
Other	2	X-CIDE 102	1.07	0.25		0.25	0.23
							350.01

Given the experimental and field data, the pressure loss and equivalent circulating density was calculated using Bingham Plastic and Power Law models for the calcium carbonates weighting agent [5], [7], [9].

1. **Bingham Plastic Model**
  $$µ=\theta_{600}-\theta_{300}=62–47=15\;$$
  $$\tau_{Y}=\theta_{300}-µ=47-15=32$$
  a. Pressure Loss along Drill Pipe in CASING
    $$V=\frac{q}{2.448(d_{2}^{2}-d_{1}^{2})}=\frac{854}{2.448(17.570^{2}-5.5^{2})}=\frac{854}{2.448(278.455)}=\frac{854}{681.6576}=1.2528\;\mathrm{ft}/\sec$$
    $$\frac{\mathrm{dP}}{\mathrm{dL}}=\frac{µV}{1000{\left(d^{2}-d^{1}\right)}^{2}}+\frac{\tau_{Y}}{200\left(d^{2}-d^{1}\right)}=\frac{15\;\times \;1.2528}{1000{\left(17.570-5.5\right)}^{2}}+\frac{32}{200\left(17.570-5.5\right)}\;=0.000128994+0.013256007=0.013385\;\mathrm{psi}/\mathrm{ft}$$
    $${∆P}_{\mathrm{drillpipes\backslash in\backslash casing}}=\mathrm{dP}/\mathrm{dL}\times \mathrm{length\backslash of\backslash drill\backslash pipe\backslash in\backslash casing}=0.013385\times \left(2074\right)=27.76049056\;\mathrm{psi}$$
  b. Pressure Loss along Drill Pipe in OPEN HOLE
    $$V=\frac{q}{2.448(d_{2}^{2}-d_{1}^{2})}=\frac{854}{2.448(16^{2}-5.5^{2})\;}=\frac{854}{2.448(225.75)}=\frac{854}{552.636}=1.5453\;\mathrm{ft}/\sec$$
    $$\frac{\mathrm{dP}}{\mathrm{dL}}=\frac{µV}{1000{\left(d_{2}-d_{1}\right)}^{2}}+\frac{\tau_{Y}}{200(d_{2}-d_{1})}=\frac{15\;\times \;1.5453}{1000{\left(16-5.5\right)}^{2}}+\frac{32}{200\left(16-5.5\right)}\;=0.000210248\;+\;0.015238095=0.015448343$$
    $${∆P}_{\mathrm{drillpipes\backslash in\backslash open\backslash hole}}=\mathrm{dP}/\mathrm{dL}\times \mathrm{length\backslash of\backslash drill\backslash pipe\backslash in\backslash open\backslash hole}=0.015448343\times 82=1.266764125\;\mathrm{psi}$$
  c. Pressure Loss along Drill Collar
    $$V=\frac{q}{2.448\left(d^{22}-d^{12}\right)}=\frac{854}{2.448\left(16^{2}-7.875^{2}\right)}=\frac{854}{2.448\left(256-62.015625\right)}=1.7854$$
    $$\frac{\mathrm{dP}}{\mathrm{dL}}=\frac{µV}{1000{(d_{2}-d_{1})}^{2}}+\frac{\tau_{Y}}{200(d_{2}-d_{1})}=\frac{15\;\times \;1.7854}{1000{\left(16-7.785\right)}^{2}}+\frac{32}{200(16-7.785)}\;=0.000396837+0.019476567=0.019873404\;\mathrm{psi}/\mathrm{ft}$$
    $${∆P}_{\mathrm{drillcollar}}=\frac{\mathrm{dP}}{\mathrm{dL}}\times \mathrm{length\backslash of\backslash drill\backslash collar}=0.019873404\times 150=2.981010597\;\mathrm{psi}$$
    $$\mathrm{Total\backslash Pressure\backslash Loss}\;∆P={∆P}_{\mathrm{drillpipes\backslash in\backslash casing}}+{∆P}_{\mathrm{drillpipes\backslash in\backslash open\backslash hole}}+{∆P}_{\mathrm{drillcollar}}=27.76049056+1.266764125+2.981010597=32.00826597\;\mathrm{psi}$$
    $$\mathrm{ECD}=MW+\frac{\Delta P}{0.052\;\mathrm{TVD}}=10.33+\frac{32.00826597}{0.052\times 2074}=9.1+0.30=9.40\;\mathrm{lbm}/{\mathrm{ft}}^{3}$$
2. **Power Law Model**
  $$n_{a}=0.657\;\log\left(\theta_{100}-\;\Theta_{3}\right)=0.657log\;\left(\frac{30}{15}\right)=0.657log\;0.2=0.657\;\times \;0.3010=0.1978K_{a}=\frac{5.11\times \Theta_{3}}{5.11^{\mathrm{na}}}=\frac{5.11\times 15}{5.11^{0.1978}}=\frac{76.65}{1.3807}=55.5141$$
  a. Pressure Loss along Drill Pipe in CASING
    $$\begin{array}{l} \;V=\frac{q}{2.448\;(d_{2}^{2}-d_{1}^{2})}=\frac{854}{2.448(17.570^{2}-5.5^{2})\;}=\frac{854}{2.448(278.455)}=\frac{854}{681.6576}=\;1.2528\;\frac{dP}{dL}=\frac{KV^{n}\left(\frac{\left(2+\frac{1}{n}\right)}{0.0208}\right)n}{144,\;000{\left(d-d\right)}^{1+n}}=55.5141\times (1.2528^{0.1978}){\left[\frac{2+\frac{1}{0.1978}}{\begin{array}{c} \frac{0.0208}{144,000{\left(17,570-5.5\right)}^{1.1978}} \end{array}}\right]}^{0.1978}\\ =\frac{55.5141\times 1.0458820837308\times 3.16574213831685}{144,00\times 19.75348705322220}=\frac{183.7550233}{2844502.136}\\ =\;0.0000646001\;\mathrm{psi}/\mathrm{ft} \end{array}$$
    $$\;\Delta P_{\mathrm{drillpipes}\;\mathrm{in}\;\mathrm{casing}}=\mathrm{dP}/\mathrm{dL}\times \mathrm{length}\;\mathrm{of}\;\mathrm{drill}\;\mathrm{pipe}\;\mathrm{in}\;\mathrm{casing}=\;0.0000646001\;\times 2074=0.133980535\;\mathrm{psi}$$
  b. Pressure Loss along Drill Pipe in OPEN HOLE
    $$\begin{array}{l} \;V=\frac{q}{2.448\;(d_{2}^{2}-d_{1}^{2})}=\frac{854}{2.448(16^{2}-5.5^{2})\;}=\frac{854}{2.448(256-30.25)}=\frac{854}{2.448\times 225.75}=\;1.5453\;\frac{dP}{dL}=\frac{KV^{n}\left(\frac{\left(2+\frac{1}{n}\right)}{0.0208}\right)n}{144,\;000{\left(d-d\right)}^{1+n}}=55.5141\;\times \;(1.5453^{0.1978}){\left[\frac{2+\frac{1}{0.1978}}{\begin{array}{c} \frac{0.0208}{144,000{\left(16-55\right)}^{1.1978}} \end{array}}\right]}^{0.1978}=\frac{55.5141\;\times \;1.08989207762134\;\times \;3.16574213831685}{144,00\;\times \;16.7169389342758}=\frac{191.5411273}{2407239.207}=\;0.0000795688\;\mathrm{psi}/\mathrm{ft} \end{array}$$
    $$\;\Delta P_{\mathrm{drillpipes}\;\mathrm{in}\;\mathrm{open}\;\mathrm{hole}}=\mathrm{dP}/\mathrm{dL}\times \mathrm{length}\;\mathrm{of}\;\mathrm{drill}\;\mathrm{pipe}\;\mathrm{in}\;\mathrm{open}\;\mathrm{hole}=0.0000795688\times 82\;=0.006524641\;\mathrm{psi}$$
  c. Pressure Loss along Drill Collar
    $$\begin{array}{l} \;V=\frac{q}{2.448\;(d_{2}^{2}-d_{1}^{2})}=\frac{854}{2.448(16^{2}-7.785^{2})\;}=\frac{854}{2.448(195.393775)}\;=\frac{854}{478.3239612}=\;1.7854\\ \frac{dP}{dL}=\frac{KV^{n}\left(\frac{\left(2+\frac{1}{n}\right)}{0.0208}\right)n}{144,000{\left(d_{2}-d_{1}\right)}^{1+n}}\;=55.5141\times (1.7854^{0.1978}){\left[\frac{2+\frac{1}{0.1978}}{\begin{array}{c} \frac{0.0208}{144,000{\left(16-7.785\right)}^{1.1978}} \end{array}}\right]}^{0.1978}\;=\frac{55.5141\;\times \;1.12146949399352\;\times \;3.16574213831685}{144,00\;\times \;12.4593573139003}=\frac{478.3239612}{1794147.453}\;=0.000108852\;\mathrm{psi}/\mathrm{ft}\\ \Delta P_{\mathrm{drillpipes}\;\mathrm{in}\;\mathrm{drill}\;\mathrm{collar}}=\mathrm{dP}/\mathrm{dL}\times \mathrm{length}\;\mathrm{of}\;\mathrm{drill}\;\mathrm{collar}\;=0.000108852\;\times \;150=0.016477796\;\mathrm{psi} \end{array}$$
    $$\begin{array}{l} \;Total\;Pressure\;Loss\;\Delta P=\Delta P_{\text{drillpipes}\;\text{in}\;\text{casing}}+\Delta P_{\mathrm{drillpipes}\;\mathrm{in}\;\mathrm{open}\;\mathrm{hole}}+\Delta P_{\mathrm{drillcollar}}\\ \;=0.133980535+0.006524641+0.016477796=0.156982973\;\mathrm{psi} \end{array}$$
    $$ECD=MW+\frac{\Delta P}{0.052\;TVD}=9.1+\frac{0.15682973}{0.052\;\times \;2074}=9.10\;{\text{lbm}/\text{ft}}^{3}$$
