# Supplementary material for: Data for analyzing drilling fluid ability to effectively achieve hole cleaning for high shear and low shear rates
Source: Data Brief. 2018 Jun 13;19:1515–21. doi: 10.1016/j.dib.2018.06.007 (PMC6141135; doi:10.1016/j.dib.2018.06.007)
Supplement: Supplementary file 1 — Supplementary material [file mmc1.docx]

All the author confirms as No conflict of Interest
